# Multisite distribution of fibrillary inclusions in a patient with light chain proximal tubulopathy: A case report

**DOI:** 10.1097/MD.0000000000039174

**Published:** 2024-08-16

**Authors:** Yingying Wang, Kai Chen, Shengguo Zhou, Wei Zhang

**Affiliations:** a Department of Nephrology, Jining No. 1 People’s Hospital, Jining, Shandong, China.

**Keywords:** fibrillary inclusions, light chain proximal tubulopathy, multiple myeloma

## Abstract

**Rationale:**

Light chain proximal tubulopathy (LCPT) is a rare form of renal impairment associated with multiple myeloma (MM). LCPT is caused by inclusions formed of free light chains that are typically crystalline, but can also be noncrystalline structures.

**Patient concerns:**

A 62-year-old man was hospitalized for the investigation of abnormal urine test results lasting for 1 year and kidney-function abnormalities persisting for more than 1 month.

**Diagnoses:**

Noncrystalline LCPT and MM.

**Interventions:**

The patient was treated with the lenalidomide, bortezomib, and dexamethasone and pomalidomide, bortezomib, and dexamethasone chemotherapy regimens.

**Outcomes:**

Complete remission of MM was achieved, and the patient’s renal function returned to normal.

**Lessons:**

This case report highlights the importance of renal pathology in the diagnosis of patients with unexplained chronic kidney disease and proteinuria.

## 1. Introduction

Light chain proximal tubulopathy (LCPT) is a rare form of kidney damage associated with multiple myeloma (MM). LCPT occurs when inclusions made of free light chains accumulate in the cytoplasm of proximal tubular cells. Free light chains impair lysosomal digestion, resulting in lysosomal abnormalities or dysfunction in the proximal tubular cells. Clinically, LCPT is characterized by reduced renal function and Fanconi syndrome; the condition progresses slowly, and evolves into end-stage renal disease in 8% to 40% of patients. LCPT manifests in 2 main histopathological forms: one with crystalline inclusions and the other with noncrystalline inclusions. In the former, free light chains (usually κ chains) typically form rhomboid or needle-shaped crystalline structures in the lysosomes and/or cytoplasm of proximal tubular cells. Three variants are described within the noncrystalline form: amyloid proximal tubulopathy, LCPT without organized deposits, and LCPT with significant fibrillary aggregations.^[1]^ In rare cases, inclusions also appear in other renal cells. The present report describes a case of myeloma-associated LCPT with noncrystalline fibrillary inclusions distributed in multiple renal cell types.

## 2. Case report

A 62-year-old man was hospitalized for the investigation of abnormal urine test results lasting for 1 year and kidney-function abnormalities persisting for more than 1 month. One year prior, a routine examination had revealed traces of blood and protein in the urine, and a serum creatinine level of 98 μmol/L; no specific treatment was administered at this time. Over a month ago, another routine examination showed microalbuminuria (161 mg/L) and elevated serum creatinine (127.6 μmol/L). The patient had a 1-year history of hypertension. The patient’s personal, marital, and family histories were unremarkable. A physical examination revealed the following: body temperature, 36.6 °C; pulse, 56 bpm; blood pressure, 120/77 mm Hg; height, 170 cm; weight, 63 kg; no rashes, superficial lymphadenopathy, or facial edema; normal cardiopulmonary auscultation; soft abdomen without tenderness or rebound tenderness; and no edema in the lower limbs.

After admission, further diagnostic tests were conducted. A urinalysis showed no traces of blood or protein, and a specific gravity of 1.008. The urinary albumin excretion rate was 47.6 mg/g of creatinine, and the 24-hours urinary protein level was 1.96 g/24 hours. The urine α1-microglobulin level was 182 mg/L. Routine blood tests showed a hemoglobin level of 136 g/L, and white blood cell and platelet counts in the normal range. Blood biochemistry revealed the following: albumin, 32.8 g/L; globulin, 34.4 g/L; urea, 7.27 mmol/L; creatinine, 126.60 μmol/L; uric acid, 173.0 μmol/L; phosphate, 0.81 mmol/L; potassium, 4.14 mmol/L; and cystatin C, 1.85 mg/L. The serum immunoglobulin levels were as follows: IgA, 0.55 g/L; IgM, <0.19 g/L; and IgG, 20.00 g/L. Serum κ light chains were measured at 7.01 g/L, and serum λ light chains were measured at 0.48 g/L, yielding a κ/λ ratio of 14.54. The C3, C4, and anti-PLA2R antibody levels were within the normal ranges. Viral assays were negative for hepatitis B and C viruses, and human immunodeficiency virus. Tests for tumor markers, immunoglobulin subtypes, antinuclear antibodies, and anti-neutrophil antibodies were all negative.

**Figure 1.**
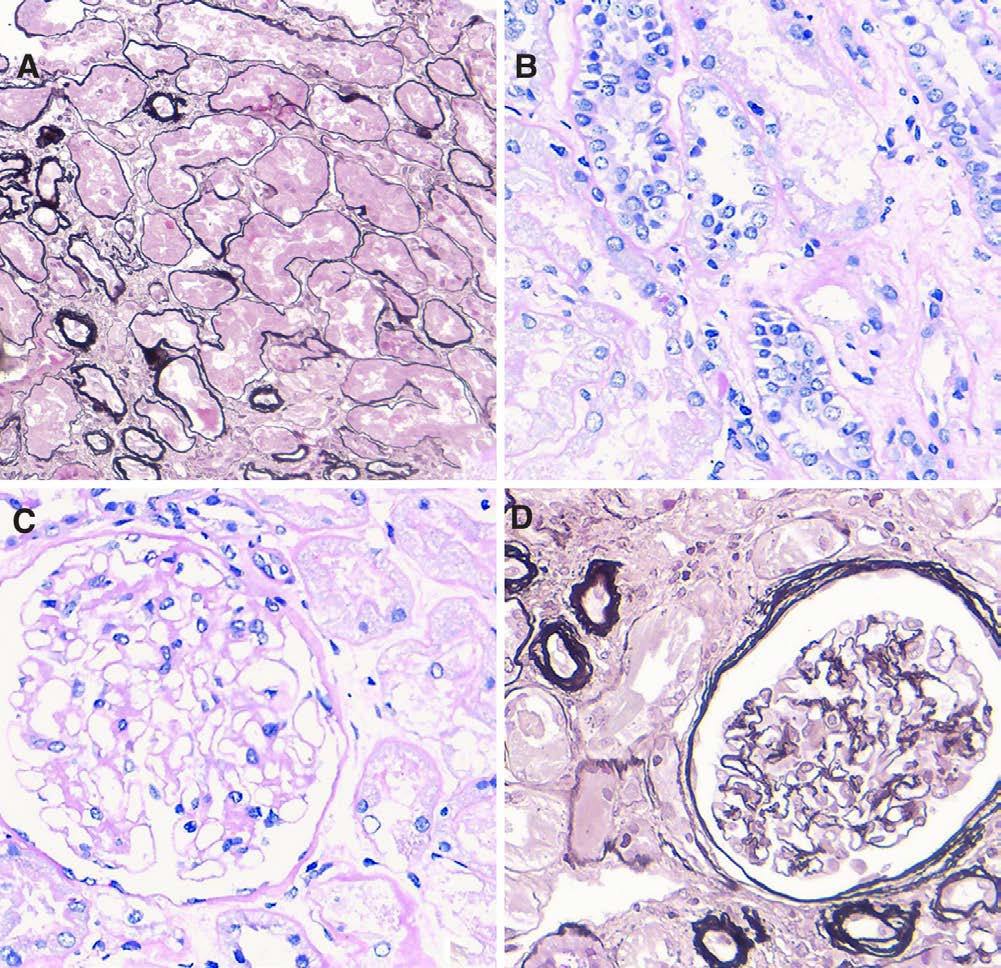
Light microscopy examination of kidney tissue. (A) Partial shedding of renal tubular epithelial cells, with exposed renal tubular basement membranes, thickening of a few tubular basement membranes, and luminal narrowing (periodic acid-Schiff-methenamine silver [PASM] staining, ×200). (B) Loss of brush borders in renal tubules, partial detachment of renal tubular epithelial cells, and enlargement and multilayered arrangement of nuclei in some renal tubular epithelial cells, without evident crystalline structures (PAS staining, ×400). (C) No obvious lesions in the glomeruli, with shedding of renal tubular epithelial cells (PAS staining, ×400). (D) Mild shrinkage of the glomerular tufts and layering of Bowman capsule walls, with shedding of renal tubular epithelial cells and tubular atrophy (PASM staining, ×400).

**Figure 2.**
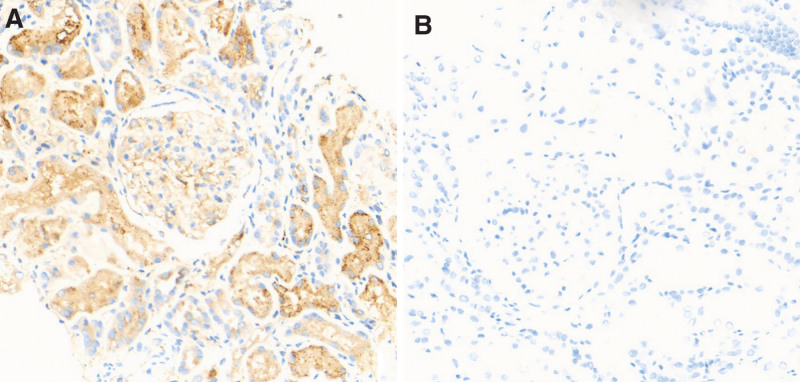
Immunohistochemical staining of kidney tissue. (A) Positive cytoplasmic staining for κ light chains in renal tubular epithelial cells (×200). (B) Negative cytoplasmic staining for λ light chains in renal tubular epithelial cells (×200).

**Figure 3.**
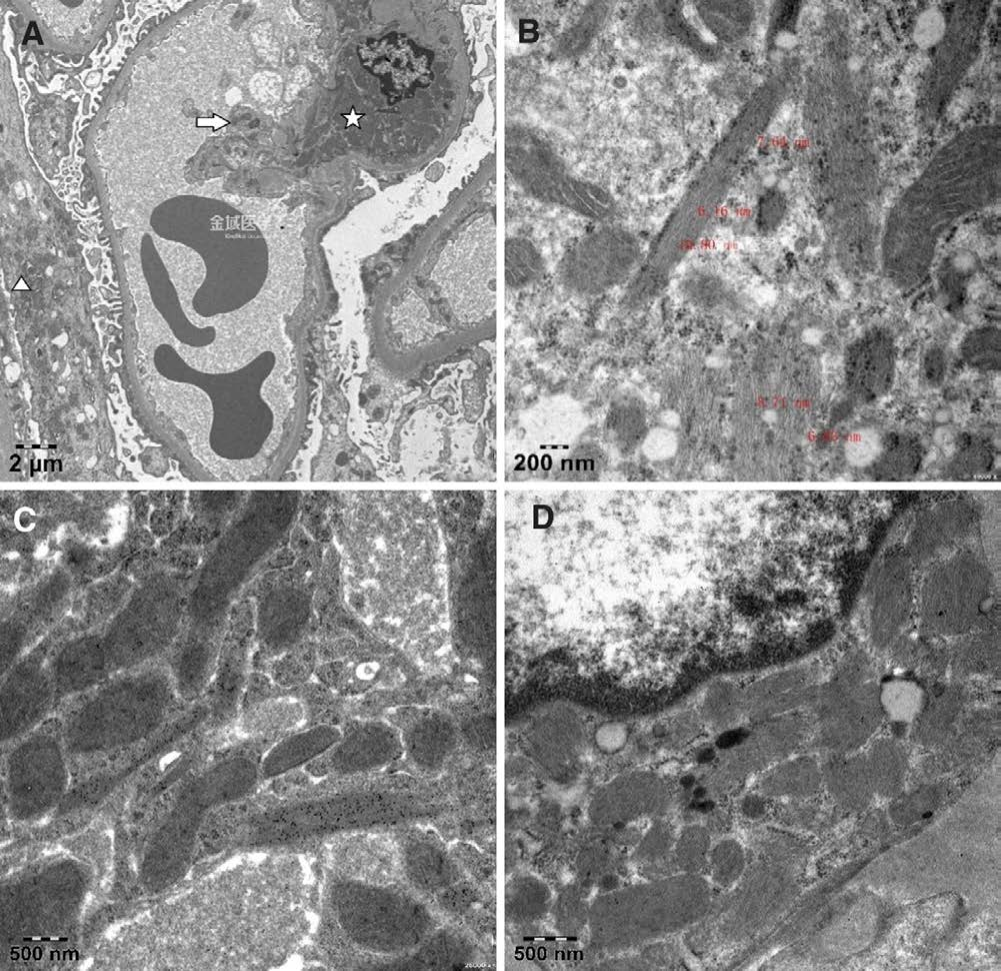
Electron microscopy and immunoelectron microscopy of renal tissue. (A) Fibrillary inclusions are distributed in mesangial cells (✩), endothelial cells (⇨), and podocytes (△). (B) The width of the fibrils within the inclusions ranges from 6 to 11 nm. (C) Immunoelectron microscopy is positive for κ chains (++, numerous colloidal gold particles are observed attached within the inclusion-like structures). (D) Immunoelectron microscopy is negative for λ chains (− to ±, minimal colloidal gold particles are found attached within certain inclusion structures).

After a thorough discussion with the patient about the benefits and risks of the procedure, we obtained consent for a kidney biopsy. Light microscopy examination of the renal cortex and medullary junction tissue revealed 39 glomeruli, with 3 showing global sclerosis; poor opening of the glomerular capillary loops; diffuse but mild segmental mesangial matrix proliferation. Tubular epithelial cell edema was observed, along with loss of the brush border in tubular epithelial cells, scattered detachment of some tubular epithelial cells, mild acute lesions (covering approximately 15% of tubular epithelial cells), and segmental thickening and atrophy of the tubular basement membrane (covering approximately 10%), with a few protein casts in the tubule lumens. Considerable chronic lymphocytic infiltration was observed in the interstitium, along with mild proliferation of the interstitial fibrous tissue and small arteriole wall thickening (Fig. 1). Immunofluorescence assays showed that the glomerular capillaries were negative for IgG, IgM, C3c, C4c, C1q, and IgA, and immunohistochemical assays revealed that the renal tissues were negative for λ chains and positive for κ chains (Fig. 2). Electron microscopy revealed no notable thickening of the glomerular basement membrane, some foot process fusion, and sparse deposits of low-density electron-dense material in select mesangial regions. A few mesangial and tubular epithelial cells contained inclusion bodies, which appeared as ordered, fine, fibrillar structures at high magnification. Immunoelectron microscopy revealed the predominant expression of κ light chains within the inclusion-like structures in a few glomerular mesangial cells, endothelial cells, podocytes, and tubular epithelial cells (Fig. 3).

In summary, the patient exhibited renal dysfunction and abnormal blood levels of light chains, with observable tubular damage under light microscopy, and expression of only κ light chains within the epithelial cells of the proximal tubules on immunohistochemistry. Immunoelectron microscopy revealed the dominant expression of κ light chains within the inclusion-like structures in a few glomerular mesangial cells, endothelial cells, podocytes, and tubular epithelial cells. The renal pathology was considered to be consistent with LCPT.

After undergoing renal biopsy, the patient was discharged due to personal reasons. We notified the patient of the renal pathology results by phone and recommended that he visit the hematology department. The patient was admitted to the hematology department for examination on August 10, 2023. Serum protein electrophoresis showed an M-spike of 19.5%. Immunofixation electrophoresis revealed a monoclonal IgGκ component in the gamma region. Blood free light chain analysis showed the following: λ light chain, 24.10 mg/L; κ light chain, 185.00 mg/L; and κ/λ ratio, 7.6763. Urine gamma immunofixation electrophoresis revealed a monoclonal IgGκ component in the gamma region. Urine free light chain tests showed a free λ light chain level of 133.00 mg/L and a free κ light chain level of 1920.00 mg/L, yielding a free κ/λ ratio of 14.4361. Bone marrow cytology showed increased proliferation in the granulocytic, erythroid, and megakaryocytic series, with a higher proportion of immature plasma cells (9% plasmacytoid ratio, with rare binucleate plasma cells). Bone marrow flow cytometry identified that monoclonal plasma cells comprised 0.89% of all nucleated cells, and were positive for CD38, CD138, cKappa, CD27, CD200, and CD56, and negative for CD19, CD45, cLambda, CD28, CD117, CD81, and CD20. Bone marrow immunohistochemical assay identified a plasma cell tumor. Bone marrow biopsy with hematoxylin and eosin, and periodic acid-Schiff staining showed relatively normal marrow proliferation (40%) and an increased number of plasma cells (approximately 15%), scattered or in small clusters. Considering all the above findings, we ultimately diagnosed this patient with noncrystalline LCPT and MM.

Four cycles of the lenalidomide, bortezomib, and dexamethasone protocol were administered. Due to renal insufficiency, the treatment was adjusted to the pomalidomide, bortezomib, and dexamethasone protocol for 3 cycles. Complete remission of MM was achieved. The serum free κ light chain level decreased to 35.9 mg/L, and the serum λ light chain level was 27 mg/L, yielding a normal serum κ/λ ratio. The renal function returned to normal.

## 3. Discussion

Monoclonal gammopathies can impact the kidney through various mechanisms, involving the glomeruli, tubules, interstitial space, and blood vessels. In LCPT, the overabsorption and buildup of monoclonal free light chains in the proximal tubular epithelium result in tubular dysfunction and the initiation of pathways that promote inflammation and fibrosis. The specific amino acid sequences and structural configurations of the variable domains of free light chains heighten their capacity to crystallize, rendering them nephrotoxic even at low concentrations.^[2]^

Our patient presented fibrillary inclusions. Previously, only 5 cases of LCPT with fibrillary inclusions have been reported. The primary disease was identified as MM in 3 cases, monoclonal gammopathy of undetermined significance in 1 case, and lymphoma in 1 case.^[3–6]^ Our patient was ultimately diagnosed with MM. Distinct from the 5 aforementioned cases, our patient had fibrillary inclusions in the tubular epithelial cells as well as in the endothelial cells, mesangial cells, and podocytes. The specific cause of inclusion formation is still unclear. There has been only 1 reported case of LCPT with abnormal accumulation of fibrillary inclusions within the glomeruli, mesangium, and tubular cytoplasm.^[7]^ Initially, we contemplated the likelihood of the patient having proliferative glomerulonephritis with monoclonal immunoglobulin deposits, but a detailed examination showed the fibrillary inclusions to be intracellular, thereby ruling out this condition. When light chains are deposited within podocytes, the condition is referred to as light chain podocytopathy, but there is currently no clear nomenclature for light chain deposition in other glomerular cells.

Immunofluorescence assays play a vital role in the diagnosis of LCPT, as they can reveal the granular distribution of deposits of individual light chain types within the tubular epithelial cytoplasm; typically, the test is positive for κ light chains and to a lesser extent, for λ light chains. However, conventional direct immunofluorescence detection of monoclonal immunoglobulins frequently encounters masking phenomena, resulting in negative findings for monoclonal light chains. The treatment of paraffin sections with protease digestion exposes antigenic epitopes, thereby increasing the sensitivity of detection.^[8]^ Using immunohistochemistry, we confirmed that in our patient, the tubular epithelial cells were κ light chain positive and λ light chain negative, showing restricted light chain expression. Furthermore, immunoelectron microscopy was used to demonstrate that the fibrillary structures within the inclusions showed κ-positive restricted expression. The Peking University First Hospital has previously reported that crystalline LCPT is predominantly caused by κ-type light chains, whereas noncrystalline LCPT is mostly associated with λ-type; the present case differs from the aforementioned pathological presentation.^[9]^

LCPT typically manifests with subacute deterioration of kidney function and mild proteinuria, frequently accompanied by aminoaciduria, glycosuria, hyperphosphaturia, and proximal renal tubular acidosis (type II), which is clinically known as Fanconi syndrome; hence, LCPT is also diagnosed as renal light chain Fanconi syndrome. This represents the quintessential clinical manifestation of LCPT. Our patient presented with renal insufficiency and proteinuria onset, with elevated urine α1-microglobulin indicating tubular damage, but without the clinical manifestations related to Fanconi syndrome. Stokes et al believe that compared to crystalline LCPT, noncrystalline LCPT is less likely to cause Fanconi syndrome; our observations are consistent with this view.^[10]^

The treatment strategies for LCPT vary based on its association with MM or other hematological malignancies. Our patient had MM-associated LCPT, and we followed the 2023 International Myeloma Working Group guidelines for managing MM-related renal impairment.^[11]^ We opted for a treatment plan that included proteasome inhibitors and immunomodulatory drugs, which were tailored to the patient’s specific situation. Following 4 cycles of chemotherapy with the cyclophosphamide, lenalidomide, and dexamethasone regimen and 3 cycles with the pomalidomide, bortezomib, and dexamethasone regimen, the patient’s serum light chain ratio and renal function were restored to normal levels.

We reiterate that the free light chains in this patient with LCPT were not limited to the tubular epithelial cells, but were also distributed in the endothelial cells, mesangial cells, and podocytes. In addition, the hematological diagnosis of this patient was unknown at the time of the kidney biopsy, and the diagnosis of LCPT via the kidney biopsy prompted a thorough examination of the bone marrow, which finally clarified the diagnosis. This highlights the importance of renal pathology in the diagnosis of patients with unexplained chronic kidney disease and proteinuria.

## Acknowledgments

We thank Medjaden Inc. for the scientific editing of this manuscript.

## Author contributions

**Conceptualization:** Yingying Wang, Kai Chen, Shengguo Zhou, Wei Zhang.

**Data curation:** Yingying Wang, Kai Chen, Shengguo Zhou.

**Formal analysis:** Yingying Wang.

**Investigation:** Wei Zhang.

**Project administration:** Yingying Wang.

**Software:** Kai Chen, Wei Zhang.

**Supervision:** Shengguo Zhou.

**Validation:** Yingying Wang, Kai Chen.

**Writing – original draft:** Yingying Wang, Kai Chen, Shengguo Zhou.

**Writing – review & editing:** Yingying Wang, Wei Zhang.
